# Geographical hotspots and correlates of early sexual debut among women in Ghana

**DOI:** 10.1186/s12978-022-01425-7

**Published:** 2022-05-12

**Authors:** Fiifi Amoako Johnson

**Affiliations:** Department of Population and Health, Faculty of Social Sciences, College of Humanities and Legal Studies, University of Cape Coast, Cape Coast, Ghana

**Keywords:** Early sexual debut, Early sexual initiation, Geographical hotspots, Maternal Health Survey, Ghana, Bayesian geoadditive semiparametric regression

## Abstract

**Objectives:**

Generalisation of sexual behaviour, including early sexual initiation, does not provide comprehensive knowledge of young people’s sexual attitudes, behaviours and challenges, given the high sociocultural diversity and economic inequalities within countries. This study examines geographical hotspots of early sexual initiation, at the district level in Ghana and the factors associated with the observed spatial patterns.

**Methods:**

Data was derived from the 2017 Ghana Maternal Health Survey, covering 21,392 women aged 15–49 years. Early sexual debut denotes first sexual intercourse before attaining the legal age of sexual consent, which in Ghana, is 16 years. The Bayesian geoadditive semiparametric regression technique was used to examine geographical hotspots and correlates of the observed spatial patterns, classified into demographic, socioeconomic and pregnancy outcome factors.

**Results:**

The results show that 26.7% (95% CI = 26.1–27.3) of women had their first sexual intercourse before attaining the age of 16 years. Hotspots of early sexual debut was observed predominantly among districts along the mainstream of the Volta Lake, which are also reported hotspots of child trafficking, labour and slavery. Demographic, socioeconomic and pregnancy related factors were identified to be correlated with the observed spatial clustering.

**Conclusion:**

Policies and interventions such as sexual and reproductive health education should target at-risk population, simultaneously addressing other child abuses perpetuating the practice.

## Plain language summary

Ghana operates a decentralised health system, where health policies and interventions, including those for sexual and reproductive health are implemented at the district level. Yet, there are no studies that systematically identify districts where sexual behaviours, such as early sexual debut, require attention. This study uses spatial models and data from the 2017 Ghana Maternal Health Survey to identify areas (districts) with high concentration of women who initiated sex before the legal age of consent. Early sexual debut refers to first sexual intercourse before attainment of the legal age (16 years) of sexual consent. Early sexual initiation has been associated with adverse sexual and reproductive health outcomes such as unwanted pregnancies and STIs. The results show that about one in four women reported having early sexual intercourse. High early sexual intercourse was observed to be particularly concentrated among districts along the mainstream of the Volta Lake. With regards to the spatial correlates, for the districts in the Oti region, high early sexual debut was associated with low educational attainment and inability to read. For those in the Bono East and Eastern regions, women who had early sexual debut were more likely to have had a miscarriage, abortion or stillbirth. Younger women, those in co-habiting relationships and those not in union were more likely to have had early sexual debut in the districts in the Ashanti, Central and Northern regions. The findings call for intensification of sexual and reproductive health education in districts along the mainstream of the Volta Lake.

## Introduction

Almost one-third of Ghana’s population are aged between 10 and 24 years, many of whom are at risk or have experienced or are experiencing the negative impacts of unwanted pregnancies and Sexually Transmitted Infections (STI), including HIV/AIDS [1, 2]. Research evidence show that early sexual debut is one of the major predisposing factors to elevated risk of STIs [2, 3]. Although there are studies that have examined the correlates of early sexual debut in sub-Saharan Africa, there are no systematic studies that identify hotspots of these practices and their spatial correlates, which are important for target interventions. Given the high sociocultural diversity and economic inequalities within countries of sub-Saharan Africa, generalisation of adolescent sexual behaviour even at the country level does not provide comprehensive knowledge of attitudes and behaviours of young people. In this regard, this study using data from the 2017 Ghana Maternal Health Survey (GMHS) examines geographic hotspots of early sexual debut (sex before the legal age of consent) among women of reproductive age in Ghana and their spatial correlates. Identifying geographical hotspots of early sexual debut and their correlates at the district level where health interventions are planned, implemented and monitored are essential for promoting targeted interventions.

The Ghana Criminal Code (amendment) Act, 2003 (Act 646) states the legal age of sexual consent as 16 years and prohibit sexual intercourse before the attainment of this age, be it consensual or not [4]. Further, the children’s Act of 1998 state the legal age of marriage as 18 years and prohibit marriage or union formation before the attainment of this age [5]. Although the laws of Ghana allow freedom of worship and customary practices, the legal age of sexual consent and marriage are the same for all. It is also worth noting that, the laws of the country are superior to any religious doctrine or cultural practices. In this regard, chapter 1(2) of the 1992 Constitution of the Republic of Ghana, states: “The Constitution shall be the supreme law of Ghana and any other law found to be inconsistent with any provision of this Constitution should, to the extent of this inconsistency, be void” [6]. It is therefore illegal in Ghana and against the sexual rights of young people to engage in sexual intercourse before attaining the legal age of 16 years.

Studies have shown that early sexual debutants are more likely to have multiple, concurrent sexual partners, transactional and unprotected sex, exposing them to acquiring STIs [7, 8]. Early sexual intercourse increases risk of unwanted pregnancy, predisposing young girls to maternal mortality and morbidity including higher risks of eclampsia, puerperal endometritis, systemic infections, low birthweight, preterm delivery and severe neonatal outcomes [9, 10]. Further, they face social challenges including limited opportunities for education and economic prospects [11]. Young people who initiate sex early are more likely to be exposed to sexual coercion, systemic physical and psychological violence [12, 13]. The effects are not only limited to the formative years of young people but also in their adult lives. It is reported that early sexual debut leads to poor psychosocial health and risky behaviours in later life, including alcoholism, drug use, antisocial and violent behaviours, low self-esteem and poor mental health, among others [14].

Early sexual initiation varies by sex and across the countries of sub-Saharan Africa. A study in five sub-Saharan African countries covering adolescents aged between 18 and 24 years revealed that in Kenya (boys = 16.3%, girls = 6.7%), Malawi (boys = 22.0%, girls = 14.1%) and Uganda (boys = 15.0%, girls = 10.4%) more boys initiate early sex compared to girls, whilst in Tanzania (boys = 6.8%, girls = 10.1%) and Nigeria (boys = 5.4%, girls = 14.5%) more girls than boys initiate sex early [15]. A multicounty study of 24 sub-Saharan African countries using nationally representative datasets, found large variations in the proportion of men and women aged 15–19 years who initiated sex before the age of 15 years, ranging from 2.0% to 27.0% for men and 5.0% to 26.0% for women [16]. The study further reported that, in Western African countries, females were more likely than men to initiate sex before age 15 years, whilst in Central, Eastern and Southern Africa a mixed pattern was observed, with early sexual initiation being more common in rural than urban areas in most countries [16]. In addition, a declining median age at first sex among young people has also been reported across many sub-Saharan African countries [2].

In Ghana, a recent study reported that 10.8% of women and 6.8% of men aged 15–24 years, reported having sex before age 15 years [17]. The 2014 Ghana Demographic and Health Survey, reported that among women age 25–49 years, 11% had their first sex by age 15 years, with a median age at first sex of 18.4 years, more than two years lower than the median age (20.7 years) at first marriage [18]. Substantial variations in median at first sexual intercourse was also reported by region (ranging from 17.6 years in the Northern region to 19.0 years in the Greater Accra region), place of residence (urban = 18.8 years, rural = 17.8 years) and wealth status (poorest = 17.6 years, richest = 19.8 years) [18]. Ethnic variations in early sexual intercourse have also been reported in Ghana, with ethnic groups (Mande, Grusi and Mole-Dagbani) in the northern part of the country initiating sex at a later age than other groups [19].

Studies have identified multifaceted factors at the individual, family and community levels that predict early sexual debut. Observed individual level predictors include low aspiration and self-esteem, lack of knowledge on sexual and reproductive health including STIs and attitudes to premarital sex [20]. At the family level, family structures and parental participation in adolescent sexual activities including supervision, sexual education, family dysfunction and socioeconomic situation are observed predictors of early sexual debut [7, 21]. In a study in Burkina Faso, Ghana, Malawi and Uganda, it was revealed that community factors such as adolescent marriage, wealth, religious affiliation, membership in adolescent social groups and use of alcohol were potential risks factors for early sexual initiation [22].

The Government of Ghana over the years has enacted a number of policies to address the sexual and reproductive health needs and challenges of young people. These include the 1999 National Youth Policy, the 2000 Adolescent Reproductive Health Policy, and the 2010 National Youth Policy of Ghana [23–25]. These policies provide comprehensive strategies for addressing the health challenges of young people, including their sexual and reproductive health needs and issue of STIs including HIV/AIDS. In 2016, the Government of Ghana enacted the 2016–2020 Adolescent Health Service Policy and Strategy to address the multiple challenges faced by young people, including their sexual and reproductive health needs and other factors that expose them to negative health and social outcomes [26]. This policy was to provide a framework for the efficient use of resources for providing health and other related services towards achieving wide-ranging health sector goals for adolescents and young people in Ghana. Despite all these policy efforts, Ghana is one of the countries in sub-Saharan Africa where young people continually face sexual, reproductive health and rights challenges [27]. Key limitations to all these policies initiatives are that they fail to identify at risk groups and geographic areas where targeted interventions are needed. Thus, identifying hotspots of early sexual debut and their spatial correlates are essential for designing and implementing target interventions to reduce its consequential negatives effects including unwanted pregnancies, maternal mortality and morbidity and STIs among adolescents and young people in Ghana.

## Methods

### Data

The data for the analysis was secondary data derived from the 2017 Ghana Maternal Health Survey (GMHS) [28]. The GMHS adopted a two-stage stratified cluster (Census enumeration areas) sampling design. A total of 900 (466 urban and 434 rural) clusters (census enumeration areas) were selected, with 30 households selected from each cluster, resulting in a total sample of 27,000 households. Within the selected households, 25,062 women aged 15–49 years were interviewed. This study covered 21,392 women for whom complete data was available. The study considered all women aged 15–49 years to examine generational changes in early sexual debut among women in Ghana. The 2017 GMHS collected detailed demographic and health information from respondents including marriage and sexual activity. The survey also collected information on the deaths of women aged 12–49 years, who died on or after 1 January 2012. The GMHS reported a median age at first sexual intercourse of 18.1 years for women aged 20–49 years, with 12.0% of these women having their first sexual intercourse by age 15 years.

Following Ghana’s Criminal Code (amendment) Act, 2003 (Act 646) [4], the outcome variable for the analysis was binary coded 1 if a woman aged between 15 and 49 years had her first sexual intercourse before attaining 16 years of age and 0 otherwise. The covariates for the analysis were selected based on evidence from the literature [2, 7, 12, 29, 30] and their availability in the GMHS data. The covariates were grouped into three categories: demographic, socioeconomic and pregnancy outcome factors to examine their associations with the spatial patterns of early sexual debut at the district level. The selected covariates, their classification and coding are shown in Table 1.

**Table 1:** Covariate selected for the analysis

Variables and their classification	Coding	Type
Demographic covariates		
Current age of respondent		Continuous
Marital status	Married = 0, Co-habiting = 1, Not in union = 2	Categorical
Parity		Continuous
Socioeconomic covariates		
Educational attainment	No formal education = 0, Middle = 1, Primary = 2, Junior High School = 3, Senior High School = 4, Higher = 5	Categorical
Religious affiliation	Catholic = 0, Protestant = 1, Pentecostal/Charismatic = 2, Other Christians = 3, Islam = 4, Traditionalist = 5, Other = 6	Categorical
Household wealth status	Lowest = 0, Second = 1, Middle = 2, Fourth = 3, Highest = 4	Categorical
Relation to household head	Head = 0, Spouse = 1, Son/daughter = 2, Son/daughter-in-law = 3, Grandchild = 4, Brother/sister = 5, Other relative = 6, Not related = 7	Categorical
Type of place of residence	Urban = 0, Rural = 1	Categorical
Reads newspaper	At least once a week = 0, Less than once a week = 1, Not at all = 2, Cannot read = 3	Categorical
Listens to radio	At least once a week = 0, Less than once a week = 1, Not at all = 2	Categorical
Watches television	At least once a week = 0, Less than once a week = 1, Not at all = 2	Categorical
Pregnancy outcome covariates		
Ever had miscarriage	No = 0, Yes = 1	Categorical
Ever had abortion	No = 0, Yes = 1	Categorical
Ever had a stillbirth	No = 0, Yes = 1	Categorical

### Statistical analysis

The Analysis of Variance (ANOVA) technique was used to examine the mean distribution of the continuous covariates (current age of respondent and parity) aggregated by respondents who had early sexual debut and those who did not have early sexual debut. The percentage distribution of respondents who had early sexual intercourse by the categorical covariates was examined, using the Chi-squared test to assess statistically significant differences. The Bayesian Geoadditive Semiparametric (BGS) regression technique was used to examine geospatial clustering of early sexual debut at the district level and the covariates associated with the observed clustering [31]. The BGS approach allows for simultaneous estimation of non-linear effects of the continuous covariates and the fixed effects of the categorical covariates in addition to the unobserved spatial effects, both spatially structured and unstructured [31]. The analysis covered the 216 districts created in 2012 and adopted for the 2017 GMHS.

The dependent variable ${y}_{ij}$ was coded 1 if woman *i* in district *j* reported having early sexual intercourse and 0 otherwise. Thus, the dependent variable follows a binomial distribution with the expected probability ${\pi }_{ij}$ of a woman having early sexual intercourse. Thus, the logistic model linking the probability ${\pi }_{ij}$ of having early sexual intercourse is of the form

1 $$y_{ij} |\eta_{ij} \sim B\left( {\pi_{ij} } \right)$$

2 $$\pi_{ij} = {\text{P}}(y_{ij} = 1|\eta_{ij)} = \frac{{{\text{exp}}\left( {\eta_{ij} } \right)}}{{1 + {\text{exp}}\left( {\eta_{ij} } \right)}}$$

where ${\eta }_{ij}$ are the covariates of interest. If we have a vector ${x}_{ij}^{^{\prime}}={({x}_{ij1},\dots ,{x}_{ijk})}^{^{\prime}}$ of *k* continuous covariates and ${\lambda }_{ij}^{^{\prime}}={({\lambda }_{ij1},\dots {\lambda }_{ijd})}^{^{\prime}}$ a vector of *d* categorical covariates, then the predictor ${\eta }_{ij}$ can be specified as

3 $$\eta_{ij} = \alpha \lambda_{ij}^{\prime } + \beta x_{ij}^{\prime }$$

where $\alpha$ is a vector of unknown regression coefficients for the categorical covariates, ${\lambda }_{ij}^{^{\prime}}$,$\beta$ is a vector of unknown regression coefficients for the continuous covariates ${x}_{ij}^{^{\prime}}$.

The BGS framework, which replaces the strictly linear predictors with flexible semiparametric predictors was adopted to account for the non-linear effects of the continuous covariates and their spatial correlation with the outcome variable. The model is thus specified as

4 $$\eta_{ij} = \alpha \lambda_{ij}^{\prime } + f_{k} x_{ijk}^{\prime } + f^{{spat\left( {S_{i} } \right)}}$$

where ${f}_{k}(x)$ are the non-linear smoothing function of the continuous variables ${x}_{ijk}$, and ${f}^{spat({S}_{i})}$ accounts for unobserved spatial heterogeneity at district *j* (*j* = 1, …, S), of which some may be spatially structured (correlated) and others unstructured (uncorrelated). The spatially structured effects show the effect of location by assuming that geographically close areas are more similar than distant areas, whilst the unstructured spatial effect accounts for spatial randomness in the model [32]. Equation 5, in this regard, is specified as.

5 $${\eta }_{ij}=\alpha {\lambda }_{ij}^{\mathrm{^{\prime}}}+{f}_{k}{x}_{ijk}^{\mathrm{^{\prime}}}+{f}^{str({S}_{i})}+{f}^{unstr({S}_{i})}$$

where ${f}^{str}$ is the structured spatial effects, and ${f}^{unstr}$ is the unstructured spatial effects and ${f}^{spat({S}_{i})}={f}^{str}+{f}^{unstr}$. The spatially structured effects depict the extent of clustering of early sexual intercourse at the district level and the associative effects of unaccounted covariates, which may be spatially clustered or random [32]. The smooth effects of continuous factors are modelled with P-spline priors, whilst the spatial effects are modelled using Markov random field priors.

The posterior modes of the structured spatial effects and their corresponding probabilities at 95% nominal level were used to examine spatial correlates of the outcome variable at the district level. The posterior probabilities at the 95% nominal level show districts where early sexual debut was statistically significantly high. That is, high positive and statistically significant estimates of the posterior mode show clustering of districts with high early sexual debut.

The estimated posterior mode of the spatial effects characterises unexplained spatially correlated covariate information. Thus, a sequential modelling approach was used to identify districts where the demographic, socioeconomic and pregnancy outcome covariates were spatially associated with the observed clustering of high early sexual debut. Model 0 was a null (constant) model. Model 1 accounted for only the spatial effects. Model 2 included the demographic covariates; Model 3 added the socioeconomic factors and Model 4 further added the pregnancy outcome predictors. Only covariates significant at *p* < *0.05* were retained in the model. The statistical software R was used for the analysis [33].

## Results

### Bivariate analysis

Table 2 shows the weighted percentage distribution of respondents who had first sexual intercourse before the legal age of 16 years by the demographic, socioeconomic and pregnancy outcomes covariates. Overall, 26.7% of women reported having their first sexual intercourse before attaining the age of 16 years. With regards to the demographic covariates, a significantly (p = 0.000) higher percentage of co-habiting women (32.5%, 95% CI = 31.0–33.9) and women not in union (27.6%, 95% CI = 26.5–28.7) reported having early sexual debut when compared to those currently married (24.0%, 95% CI = 23.2–24.8). The mean current age for women who had their first sexual intercourse before attaining age 16 years is 30.5 years (95% CI = 30.3–30.8), whilst that for those who did not have early sexual debut is 31.8 years (95% CI = 31.7–32.0). This suggests that early sexual initiation is more common among younger women compared to their older counterparts. On parity, the estimates show that women who initiate sex early are more likely to have had a higher number of children (mean = 3.2, 95% CI = 3.1–3.3) when compared to those who initiate sex after attaining the legal age (mean = 2.4, 95% CI = 2.4–2.4).

**Table 2:** Weighted distribution of respondents who had early sexual debut by background characteristics

	% [95% CI]	n	p-value
Background characteristics			
Overall	26.7 [26.1–27.3]	21,392	
Categorical covariates			
Demographic factors			
Marital status			
Married	24.0 [23.2–24.8]	10,864	0.000
Co-habiting	32.5 [31.0–33.9]	4180	
Not in union	27.6 [26.5–28.7]	6348	
Socioeconomic factors			
Educational attainment			
No formal education	32.8 [31.7–34.0]	6375	0.000
Middle	38.4 [36.7–40.1]	3269	
Primary	23.1 [20.3–26.0]	847	
Junior High School	27.1 [26.0–28.2]	6070	
Senior High School	13.7 [12.5–14.9]	3242	
Higher	5.2 [4.1–6.3]	1589	
Religious affiliation			
Catholic	26.6 [25.0–28.3]	2802	0.000
Protestant	23.5 [21.7–25.3]	2077	
Pentecostal/Charismatic	27.4 [26.4–28.4]	7667	
Other Christians	29.0 [27.2–30.8]	2533	
Islam	23.7 [22.5–24.9]	5156	
Traditionalist	37.5 [33.6–41.5]	570	
Other	35.9 [32.1–39.8]	587	
Household wealth status			
Lowest	34.6 [33.4–35.8]	5800	0.000
Second	31.2 [29.9–32.5]	4657	
Middle	28.7 [27.3–30.1]	4035	
Fourth	22.7 [21.4–24.0]	3702	
Highest	17.8 [16.5–19.1]	3198	
Relation to household head			
Head	26.0 [24.7–27.3]	4204	0.000
Spouse	26.4 [25.6–27.2]	10,669	
Son/daughter	26.3 [24.8–27.8]	3515	
Son/daughter-in-law	27.8 [24.4–31.2]	669	
Grand child	37.5 [32.8–42.2]	416	
Brother/sister	26.9 [23.1–30.7]	528	
Other relative	27.8 [25.2–30.4]	1105	
Not related	27.6 [22.4–32.8]	286	
Type of place of residence			
Urban	21.5 [20.7–22.3]	10,600	0.000
Rural	31.9 [31.0–32.7]	10,792	
Categorical covariates			
Reads newspaper			
At least once a week	12.4 [10.3–14.4]	1019	0.000
Less than once a week	14.7 [13.1–16.4]	1744	
Not at all	20.8 [19.8–21.8]	6498	
Cannot read	32.8 [32.0–33.7]	12,131	
Listens to radio			
At least once a week	24.0 [23.2–24.8]	10,145	0.000
Less than once a week	27.1 [25.9–28.3]	5328	
Not at all	31.1 [29.9–32.2]	5919	
Watches television			
At least once a week	23.2 [22.4–24.0]	11,093	0.000
Less than once a week	26.8 [25.4–28.2]	3949	
Not at all	32.9 [31.7–34.0]	6350	
Pregnancy outcome factors			
Ever had miscarriage			
Yes	28.0 [26.6–29.5]	3908	0.041
No	26.4 [25.8–27.1]	17,484	
Ever had abortion			
Yes	32.5 [31.0–34.0]	3700	0.000
No	25.5 [24.9–26.2]	17,692	
Ever had a stillbirth			
Yes	29.6 [26.9–32.2]	1126	0.029
No	26.6 [26.0–27.2]	20,266	
Continuous covariates			
Demographic factors			
Current age of respondent			
Had early sexual debut	30.5 [30.3–30.8]	5718	0.000
No early sexual debut	31.8 [31.7–32.0]	15,674	
All	31.5 [31.4–31.6]	21,392	
Total number of births			
Had early sexual debut	3.2 [3.1–3.3]	5718	0.000
No early sexual debut	2.4 [2.4–2.4]	15,674	
All	2.6 [2.6–2.6]	21,392	

n sample size

Considering the socioeconomic covariates, generally, the proportion who had early sexual debut decreases with increasing levels of educational attainment (Table 2). The estimates also shows that a significantly (p = 0.000) higher proportion of women of traditional believe initiated sex before age 16 years when compared to women of other beliefs. Further, women from poorer households were significantly (p = 0.000) more likely to have initiated sex early compared to those from richer households. Table 2 shows that women who are grandchildren to the head of the household were more likely to have initiated sex early compared to other relations. With regards to place of residence, women from rural areas (31.9%, 95% CI = 31.0–32.7) were significantly (p = 0.000) more likely to have had early sexual intercourse compared to their urban (21.5%, 95% CI = 20.7–22.3) counterparts. On source of information, Table 2 show that a higher proportion of women who do not read newspapers and those who cannot read, those who do not listen to radio and those who do not watch television, initiated sex early when compared to those who read newspapers, listen to radio or watch television at least once a week. With respect to pregnancy outcomes, the results show that women who initiated sex early were more likely to have had a miscarriage, abortion or stillbirth (Table 2).

### Geographical hotspots of early sexual debut

**Fig. 1:**
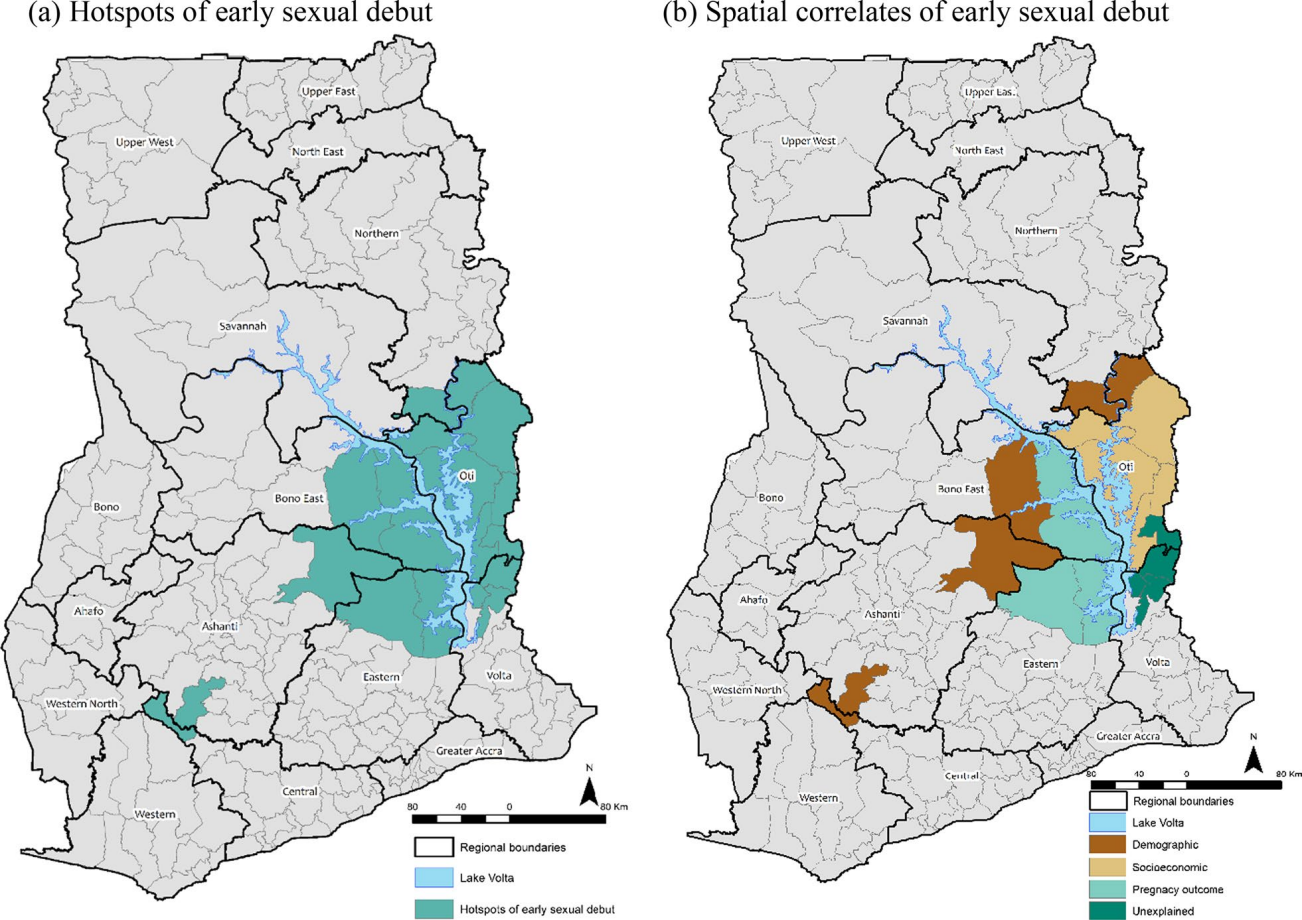
Geographical (**a**) hotspots of early sexual debut and (**b**) their associated covariates

The estimated posterior variance of the continuous covariates and the posterior odds ratios of the categorical covariates for a woman having early sexual intercourse and their corresponding 95% credible intervals are shown in Table 3, along with their model summary statistics. Interpretation of the model coefficients is based on the final model (Table 3, Model 4). The estimated deviance and AIC for Model 0 (null model) were 24,838.3 and 24,840, respectively (Table 3). When the spatial effects were included in the model (Model 1), the deviance and AIC reduced by 1039.9 and 755.0, respectively. The high reduction in the deviance and AIC after the spatial effects were included in the model indicates spatial clustering of early sexual intercourse at the district level. Figure 1a show districts where the posterior mode of the structured spatial effects was high, positive and statistically significant (clusters of high early sexual debut), at the 95% nominal level. The figure shows clustering of high early sexual debut in 19 districts. The observed hotspot districts are predominantly in the Oti region (Biakoye, Jasikan, Krachi East, Krachi West, Nkwanta South, Nkwanta North and Kadjebi districts) and Volta region (Kpando and Hohoe Municipals, Afadzato South and Krachi Nchumuru districts). Also, districts in the Bono East region (Sene West and Sene East), the Ashanti region (Amansie Central and Sekyere Afram Plains North districts), Eastern (Kwahu Afram Plains North and Kwahu Afram Plains South), Northern region (Kpandai district) and Central region (Upper Denkyira West district). An interesting spatial pattern observed was that, apart from the Upper Denkyira West district in the Central region and the Amansie Central district in the Ashanti region, all other districts with high early sexual debut were clustered along the mainstream of the Volta Lake (Fig. 1a).

**Table 3:** Posterior odds ratios of the categorical covariates and their 95% credible intervals, variance of the non-linear covariates and the spatial effects at the district-level and model summary statistics

	Model 1	Model 2 POR [95% CI]	Model 3 POR [95% CI]	Model 4 POR [95% CI]
Background characteristics				
Demographic factors				
Marital status				
Married		1.00	1.00	1.00
Co-habiting		1.55 [1.41–1.70]**	1.49 [1.35–1.63]**	1.43 [1.30–1.57]**
Not in union		1.68 [1.53–1.85]**	1.69 [1.53–1.86]**	1.69 [1.54–1.87]**
Socioeconomic factors				
Educational attainment				
No formal education			1.00	1.00
Middle			1.03 [0.93–1.14]	0.97 [0.88–1.08]
Primary			0.88 [0.72–1.08]	0.83 [0.67–1.01]
Junior High School			0.66 [0.59–0.74]**	0.61 [0.54–0.68]**
Senior High School			0.46 [0.39–0.55]**	0.44 [0.37–0.52]**
Higher			0.30 [0.23–0.39]**	0.30 [0.23–0.39]**
Reads newspaper				
At least once a week			1.00	1.00
Less than once a week			0.87 [0.67–1.13]	0.86 [0.66–1.12]
Not at all			1.01 [0.81–1.28]	1.00 [0.80–1.27]
Cannot read			1.26 [1.01–1.61]*	1.27 [1.01–1.63]*
Pregnancy outcome factors				
Ever had miscarriage				
Yes				1.36 [1.25–1.49]**
No				1.00
Ever had abortion				
Yes				2.10 [1.91–2.30]**
No				1.00
Ever had a stillbirth				
Yes				1.18 [1.03–1.37]**
No				1.00
Variance of non-linear effects				
Demographic factors				
Current age of respondent		1.0224	0.9513	0.9736
Total number of births		0.2220	0.1479	0.1560
District level variance				
Structured spatial effects (SSE)	0.1039	0.0908	0.1228	0.1114
Unstructured spatial effects (USE)	0.1305	0.085	0.082	0.0863
% Change in SSE				
Summary statistics				
Deviance	23,798.4	21,420.1	21,025.4	20,727.2
AIC	24,085	21,721.1	21,339.6	21,048.9
BIC	25,231.6	22,920.6	22,592.0	22,331.2
GVC	1.1276	1.0156	0.9975	0.9837
Change in deviance	1039.9	2378.3	394.7	298.2
Change in AIC	755.0	2363.9	381.5	290.7
Change in BIC	–	2311.0	328.6	260.8

Model 0 Deviance = 24,838.3, AIC = 24,840; **p < 0.01, *p < 0.05

### Geospatial correlates of early sexual debut

**Fig. 2:**
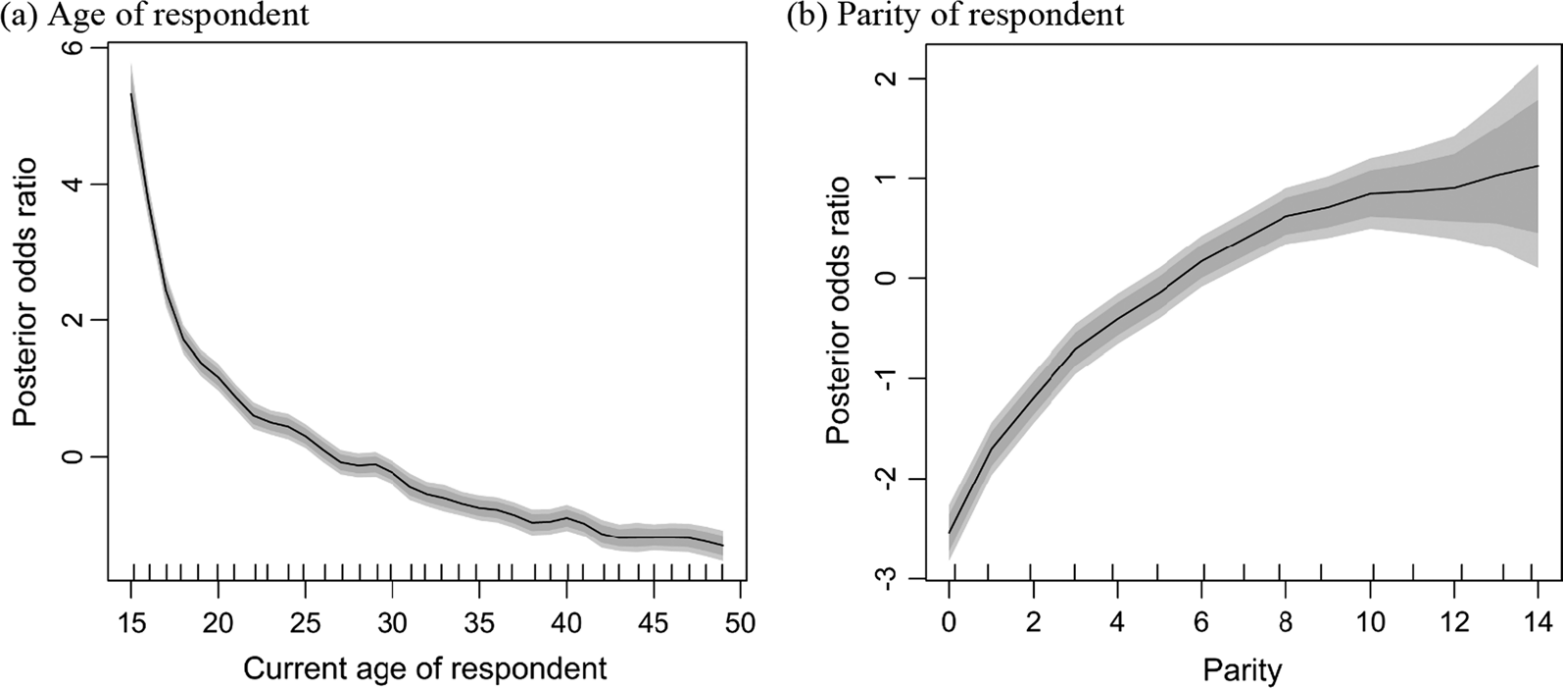
Posterior odds ratio of a woman having early sexual debut by the continuous covariates (non-linear effects)

The demographic covariates were included in Model 2 (Table 3), leading to a reduction of 2363.9 and 2311.0 in the AIC and BIC, respectively. The considerable decline in the AIC and BIC suggest that the demographic factors are spatially associated with the odds of a woman having an early sexual debut. The current age of the respondent and parity were observed to have a non-linear association with the observed spatial clustering of early sexual debut (Fig. 2a and b). Figure 2a shows higher odds of having early sexual intercourse for younger women which decreases exponentially with increasing age. This indicates that the younger generation have higher odds of engaging in early sexual intercourse when compared with the older generation. With regards to parity, Fig. 2b shows that women who had early sexual debut have higher odds of being of higher parities. The figure shows an increasing exponential relationship between the odds of having early sexual debut and women’s parity, indicating that women who had early sexual debut are more likely to have higher number of children. Further, marital status was observed to be statistically significantly associated with the probability of a woman having an early sexual intercourse. The estimates show that women who had early sexual intercourse have increased posterior odds ratio of 1.43 of being in co-habiting relationships and 1.69 of not being in union compared to those who were married. This suggest that women who had their first sexual intercourse after attaining the legal age are more likely to be married (Table 3), whilst those who had early sexual intercourse are likely to be co-habiting or not in union. The demographic factors were observed to be associated with the spatial clustering of early sexual debut in the Upper Denkyira West district in the Central region, the Amansie Central and Sekyere Afram Plains North in the Ashanti region, Nkwanta North in the Oti region, Sene West in the Bono East region and the Kpandai district in the Northern region (Fig. 1b).

The socioeconomic factors were added in in Model 3 (Table 3), leading to a decline of 381.5 and 328.6 in the AIC and BIC, respectively. The socioeconomic characteristics identified to be statistically significantly (p < 0.05) associated with the observed spatial clustering of early sexual debut were educational attainment and reading of newspapers/literacy (Table 3, Model 3). The estimates show that the higher the level of educational attainment, the low the odds of having early sexual debut. The estimated posterior odds ratios show that women with middle and primary education are not statistically significantly different from women with no formal education, however, women with junior high school (POR = 0.66, 95% CI = 0.59–0.74), senior high school (POR = 0.46, 95% CI = 0.39–0.55) and higher-level (POR = 0.30, 95% CI = 0.23–0.39) education have significantly lower odds of having early sexual debut when compared with those with no formal education. Considering frequency of reading newspapers/literacy, the results show that women who cannot read have increased odds of 27.0% of having early sexual debut compared to those who read newspapers at least once a week. This indicates that women who were illiterate were more likely to engage in early sexual intercourse. The socioeconomic factors were observed to be associated with the spatial clustering of early sexual debut in the Biakoye, Krachi East, Krachi West, Nkwanta South and Kadjebi districts in the Oti region and the Krachi Nchumuru district in the Volta region (Fig. 1b).

With regards to the pregnancy outcome covariates, the results show that women who engaged in early sexual intercourse have higher odds of having miscarriage, abortion and stillbirth. The estimated posterior odds ratios show that women who had early sexual debut are 1.36, 2.10 and 1.18 times more likely to have miscarriage, abortion and stillbirth, respectively, when compared to their counterpart who did not engage in early sexual intercourse. The pregnancy outcome covariates were associated with the observed spatial clustering of early sexual debut in the Kwahu Afram Plains North and Kwahu Afram Plains South districts in the Eastern region and Sene East in the Bono East region (Fig. 1b).

Although the Kpando and Hohoe Municipalities and the Afadzato South districts in the Volta region and the Jasikan district in the Oti region were observed among the spatial clusters of high early sexual debut, neither the demographic, socioeconomic nor pregnancy outcome covariates were significantly associated with the observed spatial clustering. Thus, the observed spatial clustering of high early sexual debut in these districts were unexplained by the selected covariates (Fig. 1b).

## Discussions

This study examined whether early sexual debut in Ghana were spatially clustered or randomly distributed at the district level and the factors that were associated with the observed spatial patterns. The findings of the study revealed that high rates of early sexual debut in Ghana was statistically significantly clustered in districts along the shores of the mainstream of the Volta Lake and its neighbouring districts, predominantly in the Oti region (Biakoye, Jasikan, Krachi East, Krachi West, Nkwanta South, Nkwanta North and Kadjebi districts) and the Volta region (Kpando and Hohoe Municipals, Afadzato South and Krachi Nchumuru districts). Also, neighbouring districts in Bono East (Sene West and Sene East districts), Ashanti (Sekyere Afram Plains North districts), Eastern (Kwahu Afram Plains North and Kwahu Afram Plains South) and Northern (Kpandai district) regions. The bordering districts of Amansie Central in the Ashanti region and Upper Denkyira West districts in the Central region were also found to form a cluster of high early sexual debut.

Insightfully, districts along the Volta Lake, where the study identified clustering of high early sexual debut, have also been reported as hotspots of child trafficking with several consequential effects for both male and female children [34]. The International Labour Organisation (ILO) estimates that more than 21,000 trafficked children engaged in hazardous activities on the Volta Lake in Ghana, which is the largest man-made lake in the world [34, 35]. Research evidence show that these areas are sated with child sexual exploitation, hazardous and abusive labour engagements, denial of medical care, education and other social opportunities [35]. Narratives from children suggest that they are not abused sexually at a young age only by their masters but also pimps who solicit their services for pay to their masters [35]. For example, it has been reported that there are hard-to-access secret island communities on the Volta Lake where trafficked girls are kept in “sexual slavery camps” to have sex with patrons whiles their masters take the money for the children’s services [36]. The observed spatial pattern of early sexual initiation conforms to stories of children trafficked and enslaved in districts along the Volta Lake, depicting that child trafficking and early sexual initiation coexist.

The socioeconomic and pregnancy outcome factors were identified to be associated with the observed spatial clustering of high early sexual debut in districts at the immediate shores of the larger stream of the Volta Lake in the Oti region (socioeconomic factors) and Eastern and Bono East regions (pregnancy outcome factors). The demographic factors on the other hand were associated with the observed pattern for districts to the further extent of the Volta Lake in the Bono East, Ashanti, Central and Northern regions. For the Upper Denkyira West district (Central region), Nkwanta North district (Oti region), Amansie Central and Sekyere Afram Plains North districts (Ashanti region), Sene West district (Bono East region) and Kpandai district (Norther region) where the demographic factors were observed to be associated with the observed spatial clustering of high early sexual debut, the results shows that younger women in these districts are more likely to have early sexual intercourse compared to their older counterparts, indicating a generation change in age of sexual initiation in these districts. Asante et al. (2018) [2] although focused on adolescents aged 15–24 years, also found that younger women have a higher odds of having early sexual debut when compared to their older counterparts. Also, women in these districts who had early sexual intercourse were more likely to be of higher parity and not be in union or in cohabiting union.

In the districts (Biakoye, Krachi East, Krachi West, Nkwanta South, Krachi Nchumuru, and Kadjebi) of the Oti region where clustering of high early sexual debut was observed, the effects were associated with the socioeconomic (education and access to information/literacy) factors. The findings show that in these districts’ women with no education or lower educational levels (primary school and below) and those who cannot read have increased odds of having early sexual debut. The literature shows that the Oti region is one of the poorest regions in Ghana, with poor infrastructure and weak institutions to promote rural development [37]. In addition, it is noted for high rates of child abuse, child labour, child abduction, teenage pregnancy and STIs including HIV/AIDS [37, 38], apprehensions concurrent with early sexual intercourse.

The study also investigated associated reproductive health problems with the observed spatial patterns of early sexual debut. The findings revealed that the clustering of high early sexual debut in the Kwahu Afram Plains North and Kwahu Afram Plains South districts in the Eastern region and the Sene East districts in the Bono East region were statistically significantly associated with the pregnancy outcome factors. In these districts, it was observed that women who engage in early sexual debut were more likely to have miscarriage, abortion and stillbirth. Studies across Ghana and sub-Saharan African have highlighted the association between early sexual debut and risk of unwanted pregnancies [2, 39, 40]. Poverty and socioeconomic deprivation have been identified to perpetuate young girls to early sexual initiation and intergeneration sex, leading to unwanted pregnancies and inevitably abortions and other unfavourable pregnancy outcomes such as miscarriages and stillbirths [40–43], as identified in the Kwahu Afram Plains North and Kwahu Afram Plains South districts in the Eastern region and the Sene East district in the Bono East region.

The findings of this study show that early sexual debut in Ghana is geographical clustered with clustering of high rates in areas where child trafficking, child labour and other related child abuses are also high. In this regard, policies and interventions such as sexual and reproductive health education should target at-risk populations, simultaneously addressing other child abuses which perpetuate the act.

## Conclusions

The study examined geographical hotspots of early sexual initiation among women in Ghana, and their spatial correlates at the district level where health policies are implemented and monitored, using the BGS regression technique. The findings of the study show that there are geographical hotspots of early sexual initiation among women in Ghana. The hotspots were found predominantly among districts along the mainstream of the Volta Lake, the biggest man-made lake in the world. These observed hotspots of early sexual debut have also been reported as hotspots of child trafficking, slavery, sexual exploitation and engagement in hazardous and abusive labour activities. The observed spatial patterns of this study reveals that child trafficking and early sexual initiation coexist along the Volta Lake of Ghana. Given the observed spatial patterns, sexual and reproductive health interventions should target at-risk populations, addressing other abuses such as child trafficking which perpetuate the act as outlined in the 2016–2020 Adolescent Health Service Policy and Strategy.

## Data Availability

The data that support the findings of this study are available from The DHS Program (https://dhsprogram.com/) but restrictions apply to the availability of these data, which were used under permission for the current study, and so are not publicly available. Data are however available upon request from The DHS Program or the author and with permission of The DHS Program.

## Abbreviations

GMHS: Ghana Maternal Health Survey
BGS: Bayesian geo-additive semiparametric
